# Phenotypic and Genotypic Characterization of Virulence Factors and Susceptibility to Antibiotics in *Salmonella* Infantis Strains Isolated from Chicken Meat: First Findings in Chile

**DOI:** 10.3390/ani10061049

**Published:** 2020-06-18

**Authors:** Lisette Lapierre, Javiera Cornejo, Sebastián Zavala, Nicolás Galarce, Fernando Sánchez, María Belén Benavides, Miguel Guzmán, Leonardo Sáenz

**Affiliations:** 1 Department of Animal Preventive Medicine, Faculty of Veterinary and Animal Sciences, Universidad de Chile, Santiago 8820808, Chile; jacornej@uchile.cl (J.C.); elias.szavalam@gmail.com (S.Z.); ngalarce@ug.uchile.cl (N.G.); fernando.sanchez@ug.uchile.cl (F.S.); mbbv@veterinaria.uchile.cl (M.B.B.); 2 Laboratory of Avian Pathology, Faculty of Veterinary and Animal Sciences, Universidad de Chile, Santiago 8820808, Chile; miguzman.vet@gmail.com; 3 Laboratory of Veterinary Vaccines, Department of Animal Biology, Faculty of Veterinary and Animal Science, Universidad de Chile, Santiago 8820808, Chile; leosaenz@uchile.cl

**Keywords:** *Salmonella* Infantis, meat chicken, broiler, antimicrobial resistance, virulence genes

## Abstract

**Simple Summary:**

*Salmonella* Infantis (*S.* Infantis) is a zoonotic pathogen that causes gastroenteritis in humans and animals, with poultry being its main reservoir. This pathogen has emerged over the last few decades in different countries, causing outbreaks in humans subsequent to foodborne transmission. It is important to be able to characterize this pathogen in order to establish control measures in the poultry industry. In this study, we investigated the presence of virulence genes, biofilm formation abilities, antibiotic resistance genes, and antibiotic susceptibility in *S.* Infantis. The results showed that the *S.* Infantis strains isolated from chicken meat for sale in supermarkets in Santiago, Chile are multidrug-resistant (MDR) and contain virulence genes, making them pathogenic. Thus, *Salmonella* Infantis should be under surveillance in the poultry food production chain with the aim of protecting public health.

**Abstract:**

*Salmonella* Infantis is a zoonotic pathogen that causes gastroenteritis in humans and animals, with poultry being its main reservoir. In Chile, there are no data to characterize *S.* Infantis strains in poultry production. In this study, 87 *S.* Infantis strains were isolated from chicken meat for sale in supermarkets in Santiago, Chile, and characterized according to their virulence genes, biofilm formation abilities, antibiotic susceptibility, and resistance genes. Through polymerase chain reaction or PCR, the strains were analyzed to detect the presence of 11 virulence genes, 12 antibiotic resistance genes, and integrase genes. Moreover, disc diffusion susceptibility to 18 antimicrobials and the ability to form biofilm in vitro were evaluated. Results demonstrated six different virulence gene profiles. Ninety-four percent of the strains were multi-resistant to antibiotics with weak biofilm formation abilities, 63.2% of the strains were broad spectrum β- lactam resistant, and the *bla*_CTX-M-65_ gene was amplified in 13 strains. Only 3.4% of the strains were fluoroquinolone resistant, and the *qnrB* gene was amplified in two strains. Colistin resistance was exhibited in 28.7% of the strains, but *mrc* genes were not amplified in any strain under study. The isolated *S.* Infantis strains are pathogenic and antibiotic multi-resistant, and thus, this *Salmonella* serotype should be under surveillance in the poultry food production chain with the aim of protecting public health.

## 1. Introduction

*Salmonella* is an important gastroenteric pathogen, and some serotypes of *Salmonella* enterica are considered emerging zoonotic pathogens, generating outbreaks worldwide in the human population. Animal food products, especially eggs and poultry meat, are the most common vehicles for *Salmonella* infections. It is estimated that *Salmonella* enterica gastroenteritis is responsible for about 93.8 million illness and 155,000 deaths worldwide each year, and, of these, 80.3 million cases are estimated to be foodborne, with very high associated costs [1].

Due to the suspected high correlation between salmonellosis in poultry and the number of human infections, Directive 2003/99/EC of the European Parliament and Council requires that the following five serotypes of *Salmonella* are monitored in poultry flocks: Enteritidis, Typhimurium, Virchow, Hadar, and Infantis. *Salmonella* Enterica subspecies enterica serovar Infantis (*S.* Infantis) is currently the emerging nontyphoidal *Salmonella* worldwide and is of major public health concern due to its frequent isolation in humans. Currently, it is ranked fourth among the top 10 human serovars. The EU ranked *S.* Infantis as the fourth most common *Salmonella* serovar found in human cases, with 1846 confirmed human cases in 2014 [2]. In Chile, the Public Health Institute published the *Salmonella* surveillance report of human clinical cases between 2012–2016 confirming the detection of 11,181 strains, with 1.3% of these corresponding to serovar *S.* Infantis [3]. This strain is frequently multidrug resistant (MDR) and seems to be spread successfully from broilers to humans through certain clones [4].

A variety of virulence factors have been shown to play different roles in the pathogenesis of *Salmonella* infections. These factors included flagella, capsule, plasmids, adhesion systems, and type 3 secretion systems (T3SS) encoded on the *Salmonella* pathogenicity islands SPI-1 and SPI-2 and other SPIs. *Salmonella* spp. require multiple genes for full virulence. Many of these genes are found in ‘pathogenicity islands’ in the chromosome [3].

The increasing incidence of *S.* Infantis infections may be further complicated by the development of resistance to medically important antimicrobials including penicillins, cephalosporins, and fluoroquinolones [5,6,7,8]. Extended-spectrum cephalosporin or fluroquinolone-resistant *Salmonella* have been isolated from food-producing animals and their products in many countries [8,9,10]. Many parts of the world have reported MDR *S.* Infantis strains from poultry and human sources, indicating that *S*. Infantis may be an emerging international public health problem [11,12,13,14]. In Chile, there are no data on the characterization of *S.* Infantis isolated from animal sources or from food.

The aim of this research was to characterize virulence factors, antibiotic resistance, and their associations in strains of *Salmonella* Infantis isolated from fresh chicken meat for sale in various supermarkets in Santiago, Chile.

## 2. Materials and Methods

### 2.1. Samples Procedures

*Sample size:* The minimum sample size was calculated to be 360 samples, and was obtained using the following formula, designed to estimate a sample proportion [15]: *n* = Zα2pq/L2 where “*n*” is the sampling size needed; “Zα” is the Z-value required for a confidence level = 1 − α, where “α” is the error; “*p*” is the expected prevalence; “*q*” is the complement of p; and “L” is the precision or absolute accepted error. In this study, α was set at 5%. Therefore, the confidence level was 95%, and Zα was 1.96. As no information exists about the prevalence of *Salmonella* Infantis strains in chicken meat in Santiago or Chile, the expected p was set at 50% and the precision L was set at 5%.

*Sampling:* In this study, a total of 361 samples were collected from 50 supermarkets in the city of Santiago, Chile, over the course of 2016. Once a month, we randomly selected eight packaged whole chicken carcasses without giblets, selecting at least one package from each of the eight different chicken-producing companies present in Chile. All retail chicken meat samples were individually and aseptically vacuum-packed. The chicken meat samples were transported to the laboratory at a temperature between 4 °C and 8 °C and analyzed within 24 h after collection.

### 2.2. Isolation and Serotyping of Salmonella

A screening using VIDAS *Salmonella* (Biomeriux^®^, bioMérieux SA, F-69280 Marcy l’Etoile, France) was initially performed. Afterward, only positive samples were analyzed according to ISO norm 6579: 2002 as it applies to *Salmonella* detection in food and animal feeding materials [16,17]. Two suspected colonies from each positive sample, with black centers on *Shigella* and *Salmonella* agar and Xylose Lysine Deoxycholate (SS and XLD) agar, were assumed to be *Salmonella* spp. The suspected *Salmonella* colonies were initially subjected to traditional morphological and biochemical testing including Gram staining, and the use of triple sugar iron agar slopes and API 20E strips (bioMérieux, Marcy l’Etoile, France). If the two suspected colonies were confirmed as *Salmonella* spp, one of them was selected to perform all subsequent phenotypic and genotypic analyses. After biochemical confirmation, *Salmonella* isolates were sent to the *Salmonella* National Reference Laboratory (Institute Public Health, Santiago, Chile) for serotype characterization using the Kauffman–White classification scheme [18].

### 2.3. Antimicrobial Susceptibility Testing

Resistance was assessed using the agar plate disk diffusion method (Kirby Bauer), which was performed according to the Clinical and Laboratory Standards Institute (CLSI) M100-S23 (CLSI,2013) [19]. The bacteria inoculum was standardized to 0.5 McFarland units using a nephelometer. The following antibiotics were used: Ampicillin (AMP), 10 μg; Amoxicillin–clavulanic acid (AMC), 20/10 μg; Ceftriaxone (CRO), 30 μg; Nalidixic acid (NA), 30 μg; Ciprofloxacin (CIP), 5 μg; Gentamicin (CN), 10 μg; Sulfamethoxazole/trimethoprim (SXT), 23.75/1.25 μg; Tetracycline (TE), 30 μg; Ceftiofur (EFT), 30 μg; Ceftazidime (CAZ), 30 μg; Cefadroxil (CFR), 30 μg; Streptomycin (S), 10 μg; Azithromycin (AZM), 15 μg; Enrofloxacin (ENR), 5 μg; Trimethoprim (W), 5 μg; Sulfisoxazole (SF), 300 μg; Chloramphenicol (C), 30 μg; and Fosfomycin (FOS), 200 μg. The disks were purchased from Oxoid^®^, UK. Multidrug resistance (MDR) was defined as the resistance to three or more antimicrobial classes. Strains were analyzed for critically important antimicrobials, as defined by the World Health Organization (WHO) [20]. Interpretation of the results of the *Salmonella* isolates was performed using the resistant breakpoints published by CLSI [19]. For enrofloxacin, ciprofloxacin breakpoints were used, and the values for azithromycin were analyzed based on Parry, 2015 and Martínez, 2016 [21,22]. Isolates with intermediate resistance were labeled as resistant and were added to the resistant count. The resistant isolates with a zone diameter less than or equal to the breakpoints for cefotaxime, ceftazidime, or ceftiofur were also examined to identify extended spectrum β-lactamses or ESBL production, using the phenotypic confirmatory test [23] with a cefotaxime (30 μg)-clavulanic acid disk (10 μg) or a ceftazidime (30 μg)-clavulanic acid disk (10 μg). For colistin, minimum inhibitory concentration (MIC) determination was performed in accordance with EUCAST/CLSI joint guidelines [24]. Results were interpreted according to the EUCAST breakpoints [25] (i.e., isolates with MICs of >2 mg/L were categorized as resistant). *Escherichia coli* ATCC 35218, *E. coli* 25922, *E. coli* NCTC 13846, and *Klebsiella pneumoniae* ATCC 700,603 were used as quality control strains.

### 2.4. Detection of Antimicrobial Resistance and Virulence Genes

The genomic DNA of *S.* Infantis was extracted using a commercial kit (Thermo Scientific, Waltham, MA, USA, GeneJET Genomic DNA Purification Kit). Concentration and quality of the extracted DNA was measured in a NANO-400 micro-spectrophotometer (Hangzhou Allsheng Instruments Co, Hangzhou, China). Samples with an absorbance ratio closest to the optimal range (1.8–2.0) were kept at −20 °C for further analysis. The presence of resistance genes beta-lactams (*bla*_TEM_, *bla*_NDM1_, and *bla*_CTX-M_), fluoroquinolons (*qnrB*), tetracycline (*tet(A*) and *tet(B*)), trimethoprim (*dfrA1*), and colistin (*mrc1, mrc2, mrc3, mrc4*, and *mrc5*) was detected by PCR amplification following previously described protocols [26,27,28,29,30,31,32], (Table 1). Additional antimicrobial resistance genes such as *bla*_CTX-M_ were sent to undergo gene sequencing using Macrogen^®^, Beotkkot-ro, Geumcheon-gu, Seoul, South Korea, and this data were aligned and analyzed using the Basic Local Alignment Search Tool (BLAST). Isolates were examined for the presence of class 1 and class 2 integrons using PCR amplification as described by Mazel et al., [33]. For virulotyping, a PCR-based test was performed for the identification of 11 virulence genes: *invA*, *pagK*, *spvC*, *sirA, gipA,* SEN1417, *trhH, sipA, sipO*, *sopD*, and *mgtC* [34,35,36,37,38]. The PCR reactions were performed following previously described protocols, as shown in Table 1. In general, the PCR was performed in a total volume of 25 μL containing 1 U Taq Polymerase (Invitrogen^®^, ON, Canada), 1× Taq buffer (5 mM KCl Tris-HCl, pH 8.5), 1.5 mM MgCl_2_, 0.1 mM dNTPs (Promega^®^), 1 μM forward and reverse primer (Promega^®^, Madison, WI, USA), and 1 µL of DNA. Brief incubation at 95 °C for 10 min was used as an initial denaturation step followed by 35 cycles of amplification. Each cycle consisted of a denaturation step at 95 °C for 1 min, followed by annealing for 1 min at different temperatures according to the target gene (Table 1) and elongation at 72 °C for 1 min. The final elongation step was conducted at 72 °C for 10 min. Control strains present in the previously-sequenced key genes were used, with the exception of *mcr* genes, which had to be sent for sequencing. In Table 1, primers, annealing temperatures, and the molecular weight of amplicons are shown for each analyzed gene.

### 2.5. Gel Documentation

The gels were stained with Gel Red (Invitrogen^®^, Carlsbad, CA, USA), the amplicons were resolved by agarose gel electrophoresis (1.5% or 2.0% agarose) at 120 V for one hour, and band visualization was carried out with a UV-transilluminator (Vilber Lourmat, Collegien, France). The concentration of agarose used was suitable for the expected band sizes. The stained gel was captured on a desktop computer using the Infinity^®^ software (Tallahassee, FL, USA).

### 2.6. Measurement of Biofilm Formation

Biofilm formation was measured using a semiquantitative adherence assay with 96-well tissue culture plates [39]. Plates were inoculated with 200 µL of Muller Hinton medium of each strain at a 10^9^ CFU/mL concentration. Prior to this, a growth curve was generated from the plate count and measurement of turbidity with a spectrophotometer at 550 nm. Plates were incubated at 25 °C for 72 h. Eight wells were used per strain to determine the number of viable cells from the biofilms and to measure the optical density from the formed biofilms. To do this, the biofilms were extracted by scraping the surface of the wells with a sterile swab, resuspended in MH broth, and diluted in series. Then, the samples were subjected to further 10-fold dilutions. Samples (0.1 mL) from each dilution were subsequently plated on XLD plates and incubated at 37 °C for 48 h before the former colonies were counted and expressed as CFU/mL. To measure the optical density from the formed biofilms, the culture medium was removed, and the wells were washed with phosphate buffer saline or PBS. To remove adhered material from the well walls, 200 µL of 80% ethanol and 20% acetone solution was added. Adhered bacteria were fixed with 150 μL of Bouin’s solution (7.5 mL picric acid; 2.5 mL 40% formaldehyde; 0.5 mL acetic acid) for 15 min, and washed again with PBS. Plates were air-dried and then stained with 150 μL of 0.1% (*w/v*) crystal violet for 5 min. Excess stain was removed, adhered bacteria were air-dried, and spectrophotometric measurements were taken at OD 550 nm in a 96-well plate reader and the average of at least three replicates was calculated.

### 2.7. Statistical Analysis

Antibiotic resistance was considered as a binary dependent variable (0 = nonresistant; 1 = resistant). Two correlations were established, one between the resistance profile of each antimicrobial and the virulence genes from the same strain, and another one between the resistance profiles of all antimicrobials according to their strains. For this, the Spearman test was used. The null hypothesis is based on a Spearman correlation, p(“rho”) of 0. The associations were considered significant when *p* < 0.05. All of the analyses previously described were performed using the Infostat^®^ software (Córdoba, Argentina) [40]. Significant results were graphed using the statistical software R (R Core Team, 2017).

## 3. Results

### 3.1. Percentage of S. Infantis Isolated from Broiler Meat

From a total of 361 broiler meat samples, 87 strains of *Salmonella* Infantis were isolated, corresponding to 24% of the samples. Additionally, three strains (0.8%) of *S.* Enteritidis were isolated, but they were not included in the research.

### 3.2. Antimicrobial Susceptibility Testing, Extended-Spectrum β-Lactamase Phenotyping and Polymerase Chain Reaction PCR Analysis of Resistance Genes

**Figure 1:**
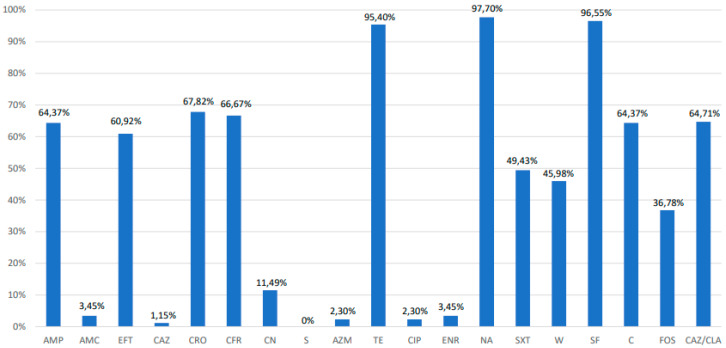
Percentages (%) of antimicrobial resistant *Salmonella* Enterica ser. Infantis. AMP, Ampicilin; AMC, Amoxicilin + Clavulanic Acid; EFT, Ceftiofur; CAZ, Ceftazidime; CRO, Ceftriaxone; CFR, Cefadroxil; CN, Gentamicin; S, Streptomycin; AZM, Azithromycin; TE, Tetracycline; CIP, Ciprofloxacin; ENR, Enrofloxacin; NA, Nalidixic Acid; SXT, Sulfamethoxazole + Trimethoprim; W, Trimethoprim; SF, Sulfisoxazole; C, Chloramphenicol; FOS, Fosfomycin; CAZ/CLA, Ceftazidime + Clavulanic Acid.

The resistance percentages for each antibiotic are shown in Figure 1. High resistance rates were detected for tetracycline (95%), nalidix acid (97%), and sulfisozaxole (96%). However, sensitivity to streptomycin (0%), ceftazidime (1.1%), ciprofloxacin (2.3%), and azithromycin (2.3%) was observed. Only one strain was found to be susceptible to all antibiotics and 94% of the strains were found to be MDR. The MDR *S*. Infantis was selected based on resistance to over three classes of antibiotics. MDR strains exhibited 31 different multi-resistance profiles (Table 2). The most common MDR profiles were: tetracycline/nalidix acid/sulfisoxazole (12 isolates); ampicillin/ceftiofur/ceftriaxone/cefadroxil/tetracycline/nalidix acid/sulfisoxazole/chloramphenicol (15 isolates); and ampicillin/ceftiofur/ceftriaxone/cefadroxil/gentamicin/tetracycline/nalidix acid/sulfamethoxazole+ trimethoprim/trimethoprim/sulfisoxazole/chloramphenicol/fosfomycin (15 isolates). When analyzing whether or not ceftazidime, ceftriaxone, and/or ceftiofur were broad spectrum β-lactamase (ESBL) producers, it was found that out of 63 strains resistant to cephalosporines, 55 were resistant to phenotypical testing (87.3%) and were classified as ESBL. Regarding colistin susceptibility, the minimum inhibitory concentration (MIC) technique was performed, where from a total of 87 analyzed strains, 25 (28.7%) strains were found to be phenotypically resistant in ranges from 4 to 8 mg/L.

Beyond the phenotypic determination, we tested for the presence of antibiotic resistance genes that could have been mediators of antibiotic resistance in *S.* Infantis. Of the three cephalosporine resistance genes, only one, *bla*_CTX-M-65_, was amplified in 13 (23.6%) of the ESBL strains. The *qnrB* gene was amplified in two (66,6%) of the three fluoroquinolone resistant strains. The *tetA* and *tetB* genes were amplified in six (7%) and 22 (25.6%), respectively, of the 86 tetracycline resistant strains, and both *tetA* and *tetB* were amplified in one (1.2%) of these strains. The *dfrA1* gene was amplified in 27 (67.5%) of the 40 trimethoprim resistant strains. The genes *mrc1, mrc2, mrc3, mrc4*, or *mrc5* were not amplified in any of the 25 colistin resistant strains. In terms of integrase genes *int* 1 and *int* 2, only gene class 1 was amplified and in low amounts, as it was found in only six (7%) of the *S.* Infantis strains analyzed.

### 3.3. Distribution of Virulence Genes among Salmonella Infantis

Six different virulotypes were found in the 87 analyzed strains of *S.* Infantis (Table 3). A total of 100% of the analyzed strains presented the genes: *inv*A; *sip*A; *sip*D and *sop*D (invasion); SEN1417; *mgt*C (intracellular survival); and *pagK* (biofilm formation), which might indicate the importance of these seven genes as virulence factors for *S.* Infantis. All 11 virulence genes analyzed were amplified in one strain, and 10 out of 11 virulence genes analyzed were amplified in four strains. The least-detected gene in the isolates was *trh*H, which codes for the putative F pilus assembly protein. This gene was amplified in only three strains.

### 3.4. Biofilm Formation

When measuring the absorbance (OD550) of the solubilized biofilms, values from 0.82 to 2.145 were reported. The data were subsequently classified according to ODc (ODc is the average optical density achieved by the negative control wells of each reading plus three standard deviations). The ODc value was 0.790. The strains were grouped into two categories according to the OD cutoff value (ODc) obtained. Overall, most of the strains exhibited low OD slightly over the threshold, and were therefore classified as weak biofilm producers. Only one strain exhibited an OD of 2.145, and was classified as a mild to strong biofilm producer.

### 3.5. Statistical Analysis

**Figure 2:**
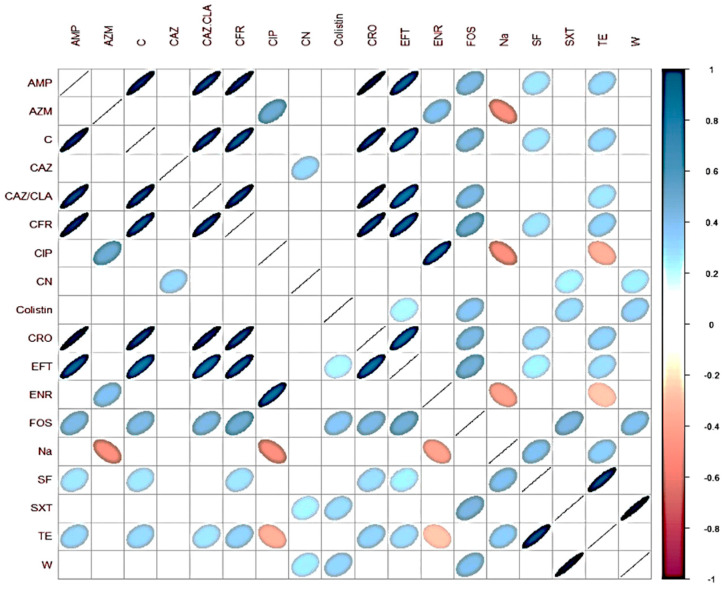
Spearman test correspondence map for phenotypic susceptibility antibiotic in strains of *S.* Infantis.

Multiple analyses were carried out for each *Salmonella* strain with data about the presence or absence of virulence genes as well as for antibiotic resistance. In this regard, a Spearman correlation coefficient greater than or equal to 0.8 or less than or equal to −0.8 implies a strong association among the analyzed variables (Austin and Sutton, 2019). A positive association was found only in antibiotic resistance. In this way, the strong associations found were: trimethoprim and sulfamethoxazole + trimethoprim (0.9331); ampicillin and ceftriaxone (0.9259); ampicillin and chloramphenicol (0.8997); ampicillin and cefadroxil (0.8994); and ceftriaxone and chloramphenicol (0.8745). Results are shown in Figure 2. Correlations among the virulence genes and resistance profiles were weak, below 0.5 for all combinations of virulence genes and the analyzed resistance profiles. Thus, there was no association between the virulence genes and antibiotic resistance.

## 4. Discussion

Salmonellosis is one of the major zoonoses that impact human and animal health worldwide, especially through global trade. In the current study, 24% of the analyzed chicken meat samples for sale in supermarkets tested positive for *Salmonella* Infantis. Only three strains of *S.* Enteritidis were isolated from the 361 samples of chicken meat, suggesting that the emergence of *S.* Infantis is replacing *S.* Enteritidis in the chicken-producing chain in Chile. The emergence of the *S.* Infantis serotype has been described in European countries and the United States [4,41,42,43,44]. In South America, it has also been described in Ecuador [45] and Peru [46]. In Brazil, *S.* Infantis has been catalogued as the second most prevalent serotype in broilers [47]. In Chile, no previous studies of *S.* Infantis in animals or in food have been conducted, and this research is the first to characterize this serotype in chicken meat for sale in supermarkets. The results suggest the presence and spread of *S.* Infantis along the food chain. These data are important to substantiate a monitoring process of the emergence of this and other serotypes of zoonotic *Salmonella* in food or food-producing animals over time.

In the *Salmonella* genus, changes in virulence gene acquisition could contribute to the increase or decrease of their virulence in the future, with human health consequences [48]. Relatedly, Brown et al. [49] characterized one *S.* Infantis strain with a multi-resistance plasmid in human patients. This strain possesses clinically important resistance associated with higher hospitalization rates. In Chile, few cases of human patients infected by *S.* Infantis have occurred [3]. However, all the analyzed strains of *S.* Infantis amplified virulence genes that would permit the infection of susceptible hosts, leading us to hypothesize that in the future, *S.* Infantis salmonellosis cases could increase in Chile.

As mentioned by Karacan Sever and Akan [50], the strains in this study showed a high prevalence of *sipA, sipD*, and *sopD* virulence genes, which facilitate the entry of *Salmonella* into the host cell through the formation of “membrane ruffling” and actin structure disruption. Karacan Sever and Akan [50] found 19 different patterns when amplifying 11 virulence genes from *S.* Infantis strains isolated from chicken, turkey, and environmental samples. This difference in the number of virulotypes expressed by *S.* Infantis (six versus 19) could be related to the diverse origin of the strains in both studies. The detection of virulence gene combinations could be a predictor of the pathogenic potential of the strain [50]. From this perspective, the bacteria analyzed in this study showed a high pathogenic potential. When observing the Spearman correlations in the studied strains, with the aim of substantiating the existence of an association between the presence of any of the virulence genes and the antibiotic resistance profiles, none of the virulence genes exhibited an association with the profile of antibiotic resistance. This could indicate that almost 100% of the strains from this study present a high number of virulence genes, given that they are antibiotic resistant.

A virulence gene found in all strains was *pagK*, which participates in biofilm formation in the *Salmonella* genus. In this regard, there are few studies about biofilm formation in *S.* Infantis strains isolated from chicken and even fewer about *S.* Infantis isolated from chicken meat [4,51,52]. In the current study, 99% of the strains were found to be weak biofilm-forming bacteria in vitro, similar to other authors’ [4,52,53] observations of weak biofilm formation in *S.* Infantis strains isolated from broiler chickens. Thus, *S.* Infantis persistence in chicken meat may not depend on a strain’s ability to form strong biofilms. In this context, the acquisition of megaplasmid pESI may confer competitive advantages in comparison to other *Salmonella* serotypes, promoting the formation of a strong biofilm [5]. Franco et al. [6] described the presence of plasmid pESI carrying the ESBL *bla*_CTX-M_ resistance gene in cefotaxime resistant *S.* Infantis strains isolated from chickens, chicken meat, and humans.

*Salmonella* resistance to β-lactams including cephalosporins due to the production of β-lactamases is considered to be of critical importance by the WHO, since transfer of resistant strains to human patients may occur through the food chain [20,54]. In Chile, the use of ceftiofur, a third-generation cephalosporin, is permitted for use in poultry, which could explain the percentages observed for cephalosporine resistance. The *bla _CTX-M 65_* gene was amplified in 13 strains, indicating that these strains are reservoirs of CTX-M ESBL genes.

The CTX-M β-lactamase lineage exhibits a striking plasticity, with many allelic variants belonging to several sublineages. This characteristic can be associated with functional heterogeneity of clinical relevance [55], indicating that further analysis of the strains that possess this gene should be carried out. Nevertheless, the presence of this gene in *S.* Infantis is of great concern, since the gene could be disseminated to other bacteria from the human intestinal microbiota. This is a worldwide problem since cephalosporin-resistant isolates of *S.* Infantis in animals and humans have recently been described in many countries [13]. All the strains in which the *bla _CTX-M_* gene was amplified were sent for sequencing, which found *bla _CTX-M 65_* to be the allelic variant. In 2014, the U.S. Food and Drug Administration found *bla _CTX-M-65_* ESBL-producing *Salmonella* Infantis in retail chicken meat [56]. Brown et al. [49] examined 29 human isolates of *S.* Infantis that carried the *bla*_CTX-M-65_ gene. Of 19 patients with travel information available, 12 (63%) reported recent travel to South America. Genetically, isolates from travelers, nontravelers, and retail chicken meat were similar. This information suggests that it may be possible for this gene to spread between countries.

Moreover, the 13 strains with the *bla*_CTX-M 65_ gene were MDR. One of these strains was also positive for the *qnrB* gene, which confers fluoroquinolone resistance. Fluoroquinolone is used as a second line treatment of invasive salmonellosis in humans and animals [57,58], and *qnr* genes have been described in Chile, Peru, Bolivia, and Colombia [59,60,61], China [62], and the United States [63], among others. Although in the current study only two strains of *S.* Infantis contained this gene, it is important to actively monitor quinolone resistance mediated by these mobile elements, in order to avoid their selection and spread. The low resistance levels to fluoroquinolones in this study could be attributed to a decrease in their use in broilers over the last few years due to current Chilean legislation that strongly discourages the use of fluoroquinolones as the first line treatment unless there is no available therapeutic alternative [64].

One last option to treat MDR-Enterobacteria in humans is the use of colistin. Twenty-five of our 87 strains were colistin resistant. Additionally, 10 colistin resistant strains also amplified the *bla*_CTX-M 65_ gene. The coexistence of ESBL and colistin resistance, currently represents a threat for public health, since both resistances could be codified in transferable plasmids [65]. None of the colistin resistant strains in this study showed amplification of transferable genes containing colistin resistance, suggesting that the determining factors for these strains could be chromosomal and non-transferable. Nevertheless, surveillance of *mcr-*like genes in zoonotic pathogen populations is necessary to understand their true impact on human health and to manage colistin use in order to minimize selection, proliferation, and spread of drug-resistant bacteria.

It has been shown that *Salmonella* is widely drug resistant and commonly multidrug resistant [66], especially with commonly used antibiotics such as tetracycline and trimethoprim. Tetracycline resistance occurs most often by the acquisition of genes that encode efflux pumps such as *tet* genes [67]. Resistance to trimethoprim in *Salmonella* is conferred by mobile resistance *dfr* genes [68]. We could not associate phenotypic resistance with resistance genes analyzed in all antibiotic resistant strains. One reason for this could be the wide variety of genes that confer resistance to tetracycline and trimethoprim, and we propose that further study is necessary.

Integrons are DNA elements that can transfer antibiotic resistance genes among bacteria, and one of the aims of this study was to identify the prevalence of class 1 and 2 integrons in *S.* Infantis strains. In our study, 7% of the strains tested positive for class 1 integrons, and 0% tested positive for class 2 integrons. This finding is in contrast to that of other authors, who have reported a higher prevalence of integrons in *S.* Infantis strains. For example, Asgharpour et al. [11] reported a class 1 integron prevalence of 36%, while Rahmani et al. [69] reported a class 1 integron prevalence of 100% in *S*. Infantis (*n* = 27) isolated from broilers. Similar findings were reported by Shahada et al. [13] in 120 strains of *S.* Infantis isolated from broiler chickens. Possibly, the strains used for the current study had other elements that confer multi-resistance such as plasmids or transposons.

Regarding the associations between antibiotic resistance among the strains, our data indicated that the resistance to certain antimicrobials was significantly associated. There were strong associations, particularly for β-lactam antibiotics. However, there were also important associations between antibiotics from different families such as sulfisoxazole and tetracycline or chloramphenicol and cefadroxil (Figure 2). Overall, tetracycline resistance is one of the most prevalent ones in zoonotic *Salmonella* due to its extended use in veterinary medicine [70], and this factor could influence its association with sulfisoxazole. Chloramphenicol use is forbidden in food producing animals in Chile, as well as in most other countries. Nevertheless, resistance to this antibiotic has been observed in Enterobacteria isolated from broilers [71]. There could be several explanations for the observed associations including the existence of linked genes within mobile elements such as plasmids, and co-selection due to the use of one of these antimicrobials in animals. In addition to the use of antibiotics in animal production, it is important to remember that environmental pollution with antibiotic waste exists in water and soil, which has caused an environmental resistome that could be transferred to zoonotic bacteria. This study highlights the possible emergence of MDR *Salmonella* Infantis in chicken meat in Chile. The findings suggest that special efforts must be made for the effective control of *S.* Infantis in food-processing environments.

## 5. Conclusions

In this study, a phenotypical and genotypical characterization of virulence factors and antibiotic susceptibility in *Salmonella* Infantis strains isolated from chicken meat available in supermarkets was carried out for the first time in Chile. Our study provides strong evidence of dissemination of virulent and MDR *S.* Infantis in this chicken meat. The strains exhibited six virulotypes with several genes that play a critical role in *Salmonella* infection pathogenesis. Moreover, these strains were also multi-resistant to antibiotics considered of great importance for human and animal health. In the strains analyzed in this study, resistance determiners associated with mobile elements such as the *bla*_CTX-M 65_ and *qnrB* genes were amplified. Some strains were found to be resistant to colistin, but none of them were positive for *mrc* genes. These findings suggest that it is important to monitor the emergence of *S.* Infantis throughout the food chain process due to its importance for public health.

## Figures and Tables

**Table 1:** Primers used to detect antimicrobial resistance and virulence genes.

Gene	Primers (5′-3′)	Product Size (pb)	Annealing T°	Reference
*bla TEM*	F: ATCAGCAATAAACCAGC R: CCCCGAAGAACGTTTTC	516	54 °C	Colom et al. (2004)
*bla CTX-M*	F: ATGTGCAGYACCAGTAARGTKATGGC R: TGGGTRAARTARGTSACCAGAAYSAGCGG	592	58 °C	Mulvey et al. (2003)
*bla NDM1*	F: CTGAGCACCGCATTAGCC R: GGGCCGTATGAGTGATTGC	621	52 °C	Pfeifer et al. (2011)
*qtetA*	F: GGTTCACTCGAACGACGTCA R: CTGTCCGACAAGTTGCATGA	577	55 °C	Ng et al. (2001)
*tetB*	F: CCTCAGCTTCTCAACGCGTG R: GCACCTTGCTGATGACTCTT	634	55 °C	Ng et al. (2001)
*dfrA1*	F: GGAGTGCCAAAGGTGAACAGC R: GAGGCGAAGTCTTGGGTAAAAAC	367	55 °C	Torkan et al. (2015)
*qnrB*	F: GATCGTGAAAGCCAGAAAGG R: ACGATGCCTGGTAGTTGTCC	469	53 °C	Robicsek et al. (2006)
*mrc1*	F: AGTCCGTTTGTTCTTGTGGC R: AGATCCTTGGTCTCGGCTTG	320	58 °C	Rebelo et al. (2018)
*mrc2*	F: CAAGTGTGTTGGTCGCAGTT R: TCTAGCCCGACAAGCATACC	715	58 °C	Rebelo et al. (2018)
*mrc3*	F: AAATAAAAATTGTTCCGCTTATG R: AATGGAGATCCCCGTTTTT	929	58 °C	Rebelo et al. (2018)
*mrc4*	F: TCACTTTCATCACTGCGTTG R: TTGGTCCATGACTACCAATG	1116	58 °C	Rebelo et al. (2018)
*mrc5*	F: ATGCGGTTGTCTGCATTTATC R: TCATTGTGGTTGTCCTTTTCTG	1644	58 °C	Rebelo et al. (2018)
*int1*	F: GGGTCAAGGATCTGGATTTCG R: ACATGGGTGTAAATCATCGTC	483	62 °C	Mazel et al. (2000)
*int2*	F: CACGGATATGCGACAAAAAGGT R: GTAGCAAACGAGTGACGAAATG	233	62 °C	Mazel et al. (2000)
*gipA*	F: ACGACTGAGCAGGCTGAG R: TTGGAAATGGTGACGGTAGAC	518	58 °C	Huenh et al. (2010)
*mgtC*	F: TGACTATCAATGCTCCAGTGAAT R: ATTTACTGGCCGCTATGCTGTTG	677	58 °C	Huenh et al. (2010)
*trhH*	F: AACTGGTGCCGTTGTCATTG R: GATGGTCTGTGCTTGCTGAG	418	53 °C	Huenh et al. (2010)
*spvC*	F: CTCCTTGCACAACCAAATGCG R: TGTCTCTGCATTTCACCACCATC	570	53 °C	Huenh et al. (2010)
*sirA*	F: TGCGCCTGGTGACAAAACTG R: ACTGACTTCCCAGGCTACAGCA	313	55 °C	Huenh et al. (2010)
*pagK*	F: ACCATCTTCACTATATTCTGCTC R: ACCTCTACACATTTTAAACCAATC	151	60 °C	Huenh et al. (2010)
*invA*	F: GTGAAATTATCGCCACGTTCGGGCAA R: TCATCGCACCGTCAAAGGAACC	284	64 °C	Malorny et al. (2003)
*SEN1417*	F: GATCGCTGGCTGGTC R: CTGACCGTAATGGCGA	670	58 °C	Pan et al. (2009)
*sipA*	F: ATGGTTACAAGTGTAAGGACTCAG R: ACGCTGCATGTGCAAGCCATC	2055	53 °C	Shah et al. (2011)
*sipD*	F: ATGCTTAATATTCAAAATTATTCCG R: TCCTTGCAGGAAGCTTTTG	1029	53 °C	Shah et al. (2011)
*sopD*	F: GAGCTCACGACCATTTGCGGCG R: GAGCTCCGAGACACGCTTCTTCG	1291	59	Raffatellu et al. (2005)

**Table 2:** Phenotypic resistance profiles detected in *Salmonella* Infantis strains.

Resistance Phenotype	No. Isolates
NA	4
ENR-NA	1
TE-NA-SF	12
CRO-TE-NA-SF	1
CFR-TE-NA-SF-FOS	1
TE-NA-SXT-W-SF	3
TE-NA-SF-C-FOS	1
CFR-CIP-NA-SXT-W-SF	1
CN-TE-NA-SXT-W-SF	4
AMC-CFR-TE-NA-SXT-W-SF	1
EFT-CN-TE-NA-SXT-W-SF	1
CFR-CN-TE-NA-SXT-W-SF	1
AMP-EFT-CRO-CFR-TE-NA-SF-C	15
AMP-CN-TE-ENR-NA-SXT-W-SF	1
AMP-CRO-TE-NA-SXT-W-SF-C	1
AMP-EFT-CRO-CFR-TE-NA-SXT-SF-C	1
AMP-EFT-CRO-CFR-CN-TE-NA-SF-C	1
AMP-EFT-CRO-CFR-TE-NA-SXT-W-SF-C	1
AMP-EFT-CRO-CFR-CN-TE-ENR-NA-SF-C	3
AMP-EFT-CRO-CFR-CN-TE-NA-SF-C-FOS	1
AMP-EFT-CRO-CFR-TE-NA-SXT-W-SF-C	1
AMP-EFT-CRO-CFR-AZM-TE-NA-CIP-SXT-SF-FOS	1
AMP-EFT-CRO-CFR-CN-TE-ENR-NA-SF-C-FOS	3
AMP-EFT-CRO-CFR-CN-AZM-TE-ENR-NA-SF-C	1
AMC-EFT-CRO-CFR-TE-NA-SXT-W-SF-C-FOS	1
AMP-EFT-CRO-CFR-TE-NA-SXT-W-SF-C-FOS	3
AMP-EFT-CRO-CFR-CN-TE-NA-SXT-W-SF-FOS	1
AMP-EFT-CRO-CFR-CN-TE-NA-SXT-W-SF-C-FOS	15
AMP-AMC-EFT-CAZ-CRO-CFR-CN-TE-ENR-NA-SF-C-FOS	1
AMP-AMC-EFT-CRO-CFR-CN-TE-NA-SXT-W-SF-C-FOS	1
AMP-EFT-CRO-CFR-CN-AZM-TE-NA-SXT-W-SF-C-FOS	1
AMP-EFT-CRO-CFR-CN-TE-ENR-NA-SXT-W-SF-C-FOS	1
AMP-EFT-CRO-CFR-CN-TE-NA-ENR-SXT-W-SF-C-FOS	1

AMP Ampicilin; AMC, Amoxicilin + Clavulanic Acid; EFT, Ceftiofur; CAZ, Ceftazidime; CRO, Ceftriaxone; CFR, Cefadroxil; CN, Gentamicin; S, Streptomycin; AZM, Azithromycin; TE, Tetracycline; CIP, Ciprofloxacin; ENR, Enrofloxacin; NA, Nalidixic Acid; SXT, Sulfamethoxazole + Trimethoprim; W, Trimethoprim; SF, Sulfisoxazole; C, Chloramphenicol; FOS, Fosfomycin.

**Table 3:** Virulence gene combinations (virulotypes) and their frequency in the isolated strains.

ID ^1^	Virulotypes: gipA-spvC-sirA-invA-SEN1417-pagK-sipA-sipO-sopD-mgtC-trhH	No. of Strains
2	11011111110	65
3	10011111110	11
6	11111111110	4
7	10111111110	4
15	11111111111	1
77	11011111111	2

^1^ ID numbers were assigned according to the virulotype appearance in the strain list.
